# A Prey–Bacterivore Interaction: CdtB Involvement in the Cytotoxicity Mechanism of *Escherichia albertii* Against *Tetrahymena thermophila* CU427

**DOI:** 10.3390/microorganisms14102317

**Published:** 2026-10-11

**Authors:** Istiana Nur Vidayanti, Kenta Watanabe, Ema Fujino, Takashi Shimizu, Masahisa Watarai

**Affiliations:** 1 The Joint Graduate School of Veterinary Medicine, Yamaguchi University, 1677-1 Yoshida, Yamaguchi 753-8515, Japan; d002tnu@yamaguchi-u.ac.jp; 2 Laboratory of Veterinary Public Health, Joint Faculty of Veterinary Medicine, Yamaguchi University, 1677-1 Yoshida, Yamaguchi 753-8515, Japan; b025tbv@yamaguchi-u.ac.jp (E.F.); watarai@yamaguchi-u.ac.jp (M.W.); 3 International Research Center for One Welfare, Joint Faculty of Veterinary Medicine, Yamaguchi University, 1677-1 Yoshida, Yamaguchi 753-8515, Japan; shimizut@yamaguchi-u.ac.jp

**Keywords:** *cdtB*, *Escherichia albertii*, host-pathogen interaction, *Tetrahymena thermophila*

## Abstract

*Escherichia albertii* is a Gram-negative, facultative anaerobic bacillus recognized as a zoonotic foodborne pathogen. Aquatic *E. albertii* populations likely coexist with protist hosts. Dynamic interactions between pathogenic bacteria and their protist predators may extend beyond shared growth spaces and involve host-directed cytotoxicity. *E. albertii* carries cytotoxicity-associated genes such as *cdt*, *stx*, and *astA.* This study aimed to elucidate the role of *cdtB* in *E. albertii* interaction within the *Tetrahymena thermophila* CU427 strain. Cytolethal distending toxins (CDTs) are tripartite protein exotoxins; CdtB requires CdtA and CdtC to bind to the glycosphingolipids ganglioside GM1 and GM3 and induce full cytotoxicity in mammalian cells. Ganglioside GM1 was highly distributed in CU427 in our study, whereas GM3 showed no comparable distribution, suggesting that ganglioside GM1 may act as a receptor for CdtB in *Tetrahymena* cells. However, cholera toxin B subunit treatment failed to inhibit *E. albertii* cytotoxicity against CU427. A LIVE/DEAD assay using Carboxyfluorescein succinimidyl ester/Propidium Iodide staining was conducted for CU427 infected with *E. coli* expressing recombinant *E. albertii* CdtB. CU427 viable cell number decreased half-fold after 24 h in a similar tendency with *E. albertii* infection. These results indicate that CdtB is involved in *E. albertii* cytotoxicity against protist hosts, particularly *Tetrahymena*, and suggest that the cytotoxic mechanism, especially its interaction with host cells, differs from that in mammalian cells. Thus, this study provides deeper insights into the involvement of CdtB in the *E. albertii* cytotoxicity mechanism.

## 1. Introduction

Frequent bacterial infection-associated human food poisoning and major concern over diarrheal disease outbreaks among children in Bangladesh contributed to the first identification of *Escherichia albertii* [1]. The *E. albertii*-associated gastroenteritis outbreak involves symptoms such as diarrhea, abdominal pain, fever, and nausea [2]. The zoonotic food- and waterborne pathogen *E. albertii* is a Gram-negative, nonmotile bacterium that is closely related to enteropathogenic *Escherichia coli* (EPEC) or enterohemorrhagic *E. coli* (EHEC). Earlier reports misidentified *E. albertii* as *Hafnia alvei* based on *eae* gene presence [3] or as *Shigella* species because of shared morphological and biochemical features [4]. *E. albertii* harbors several virulence factors, such as *stx* genes encoding Shiga toxins, with all *stx* positive *E. albertii* containing the *stx2f* and/or *stx2c* genes. The heat-stable enterotoxin 1 gene (*astA*) is frequently detected in *E. albertii* and encodes EAST-1, a toxin linked to diarrheagenic *E. coli* [5]. Cytolethal distending toxin (CDT), a heat-labile genotoxin, is produced by several Gram-negative bacteria, including *E. coli*, *Campylobacter jejuni*, *Aggregatibacter actinomycetemcomitans*, and *Salmonella enterica.* Most *E. albertii* strains also harbor *cdtB*-II/III/IV or *cdtB*-I genes [6,7]. Bacterial enterotoxins bind the monoganglioside GM1 receptor, an abundant sialic acid-containing cell membrane glycosphingolipid, to enter host cells and cause intoxication. Monosialodihexosylganglioside GM3, along with GM1, has been suggested to be a mammalian tissue receptor for CDT [8,9].

The bacterivorous *Tetrahymena thermophila* are unicellular, ciliated eukaryotes and part of the Oligohymenophorea class and are 30–50 µm in length [10]. *T. thermophila*, a common soil and freshwater ecosystem inhabitant, may serve as a pathogenic-bacteria vector—that is, *Legionella pneumophila* [11], *Listeria monocytogenes* [12], *E. coli* [13], and diarrheagenic *E. coli* [14]. *T. thermophila* may facilitate waterborne pathogen transmission as a Trojan-horse factor, including *E. albertii* transmission. On the other hand, some *Legionella* strains show cytotoxicity against protist host cells and actively reject host association [11,15]. The establishment of relationships between parasitic bacteria and their protist hosts is extremely complicated, and it is likely that they have mechanisms for selecting the optimal host–bacteria combination. Consequently, the given mechanism of host–pathogen interaction in the environment may serve as a key factor for zoonotic prevention. Thus, this study evaluated how *E. albertii* and *T. thermophila* establish or control an in vitro prey–predator relationship through CdtB roles by assessing the relative survival of both organisms.

## 2. Materials and Methods

### 2.1. Bacterial and Protist Strains and Growth Conditions

*E. albertii* strain GTC16441, a clinical origin, was obtained from the National Bioresource Project (NBRP) Pathogenic Bacteria (https://pathogenic-bacteria.nbrp.jp/en/ (accessed on 13 July 2026)). *E. albertii* and *E. coli* strain DH5α (Takara Bio, Shiga, Japan) were maintained as 20% glycerol stocks at −80 °C and cultured at 37 °C on Luria Bertani (LB) (Miller, Nacalai tesque Inc., Kyoto, Japan) with or without 1.5% agar. Ampicillin (100 µg/mL) was used when required. To induce protein expression, isopropyl-β-D-thiogalactopyranoside (1 mM) was added to LB. *T. thermophila* strain CU427 was cultured and maintained in sucrose, proteose peptone medium (1.5% proteose peptone, 0.5% yeast extract, 0.5% glucose) (Beckton, Dickinson and Company, New Jersey, NJ, USA) with 33 µM FeCl_3_ supplementation at 30 °C. *T. thermophila* CU427 was passaged regularly every two to three days. *Paramecium caudatum* strain dKNZ-12O, which belongs to a similar ciliate class, Oligohymenophorea, was used as a comparative control. It was cultured at 25 °C using 2.5% fresh lettuce juice in Dryl’s solution [15]. Protist cells used in this study were provided by NBRP Paramecium (http://nbrpcms.nig.ac.jp/paramecium/ (accessed on 13 July 2026)).

### 2.2. Infection Assay Screening for Paramecium and Tetrahymena with E. albertii

To uncover *E. albertii* cytotoxicity toward *Paramecium* and *Tetrahymena*, we performed an infection assay using various multiplicity of infection (MOIs 10, 100, and 1000) at designated incubation temperatures (25 °C for *Paramecium* and 30 °C for *Tetrahymena*). The number of viable cells with normal morphology and motility was quantified using microscopy at 48 h postinfection (hpi). The *E. coli* DH5α strain served as a control.

### 2.3. Confirmation of Cytotoxicity-Related Genes and Fold Change Expression in E. albertii

We screened the presence of genes encoding several toxins in *E. albertii* using set primers of *cdt*-F, *cdt*-R; *cdtIB*-F, *cdtIB*-R; *cdtIVB*-F, *cdtIVB*-R; *stx*-F, *stx*-R; *stx2f*-F, *stx2f*-R; *elt*-F, *elt*-R; *est*-F, *est*-R; and *astA*-F, *astA*-R (Table 1) [16]. For *cdtB* and *astA*, we continued to measure their gene expression using quantitative real-time PCR. RNA was extracted from overnight *E. albertii* cultures grown at 25 °C, 30 °C, and 37 °C using TRIzol reagent (Thermo Fisher Scientific, Waltham, MA, USA), then reverse-transcribed into cDNA using ReverTra Ace qPCR RT Master Mix with gDNA Remover (Toyobo, Osaka, Japan) following the manufacturer’s instructions. Quantitative assays were performed on each cDNA with the Thunderbird SYBR qPCR Mix (Toyobo) using an Applied Biosystems StepOne Real-Time PCR System. 16s rRNA genes were used as endogenous controls to normalize all data. The relative expression levels of the genes of interest were calculated using the relative quantification method (∆∆C_T_), where each culture temperature was compared to the level when *E. albertii* was cultured at 37 °C (defined as 1).

### 2.4. DNA Recombination

The targeted region of *cdtB* of *E. albertii* was amplified using KOD DNA Polymerase (Toyobo) with CdtB for pCold primers (Table 1) and ligated into *Sal*1-cut pCold using In-Fusion cloning enzyme (Takara Bio). The successfully cloned samples were transformed into *E. coli* DH5α on LB containing 100 µg/mL of ampicillin. The cell lysates were separated on 10% polyacrylamide gels and stained with EzStain Aqua (ATTO Corporation, Tokyo, Japan). To assess the growth rate of successful recombinant bacteria, we performed a growth culture analysis with OD_595_ in triplicate.

### 2.5. Confirmation of GM1 and GM3 Ganglioside Expression on Paramecium and Tetrahymena and Inhibition Test

*P. caudatum* and *T. thermophila* were applied to a glass slide to evaluate the expression of ganglioside GM1 and GM3 (Matsunami Glass, Osaka, Japan). Samples were air-dried to attach the cells to the glass surface and then fixed with 4% paraformaldehyde (PFA) in PBS for 10 min at 25 °C. Subsequently, samples were washed twice with PBS and incubated with Cholera toxin B subunit (CTxB) conjugated with Alexa Fluor 594 (Molecular Probes, Eugene, OR, USA) at a concentration of 1 µg/mL and anti-GM3 antibody (Tokyo Chemical Industry, Tokyo, Japan) at a concentration of 10 µg/mL for 1 h. TRITC-conjugated anti-mouse IgG (Proteintech, Rosemont, IL, USA) was used as the 2nd antibody for the anti-GM3 antibody. The distribution of ganglioside GM1 and GM3 expression was observed through a FluoView FV 1000 confocal laser microscope (Olympus, Tokyo, Japan). In the inhibition test, *T. thermophila* CU427 was infected with *E. albertii* at MOIs 0, 10, and 1000 with either CTxB or anti-GM3 antibody at concentrations of 0, 0.1, 1, and 5 µg/mL, or with both CTxB and anti-GM3 antibody at a concentration of 5 µg/mL. The viable cells were counted at 24 hpi using the same method as described above.

### 2.6. Evaluation of Recombinant CdtB Expressed in E. coli for T. thermophila

*E. albertii*, *E. coli* pCold (empty-vector control), and *E. coli* pCold_*cdtB* were each cocultured with *T. thermophila* CU427 at 30 °C using an MOI of 100. Ampicillin 100 µg/mL and IPTG 1 mM were used for recombinant strain to induce protein expression. The viable cells were counted at 24 hpi using the same method as described above.

### 2.7. LIVE/DEAD Assay of T. thermophila Using CFSE/PI Double Staining

To determine the live/dead number of *T. thermophila* cells, Carboxyfluorescein succinimidyl ester (CFSE) and Propidium Iodide (PI) double staining were used following the manufacturer’s protocols (Dojindo, Kumamoto, Japan) with a certain incubation time modification. *T. thermophila* cultures infected with *E. albertii*, *E. coli* pCold_*cdtB*, and *E. coli* pCold (empty-vector control) at an MOI of 100 were washed once with PBS at 0.5 and 24 hpi. Next, the pellet was resuspended in 1 mL PBS. CFSE stock solution was prepared immediately before use by adding 2 μL of 1 mg/mL CFSE in DMSO to 1 mL PBS, followed by the addition of 20 μL stock solution to 1 mL *T. thermophila* culture in PBS. CFSE-stained cultures were incubated at 30 °C for 15 min, followed by one PBS wash. For PI staining, 1 μL of 1 mg/mL PI solution was added to 1 mL CFSE-stained *T. thermophila* culture and incubated at room temperature, under the dark, for 5 min, followed by PFA fixation at a final concentration of 4%. Finally, 10 μL stained *T. thermophila* suspension from the tube bottom was mounted on a glass slide, fixed with a circular coverslip and nail polish, and air-dried. The prepared slides were subsequently examined under a FluoView FV1000 confocal laser scanning microscope (Olympus).

### 2.8. Data Analyses

All experiments were conducted at least two times using duplicate or triplicate samples. Results are presented as mean ± SD, and statistical analyses were performed using Prism 10 software (GraphPad, La Jolla, CA, USA). One-way ANOVA was used to analyze the *Tetrahymena* fold change number using various bacterial strains (*E. albertii*, *E. coli* pCold, *E. coli* pCold-*cdtB*) at 24 hpi. Furthermore, two-way ANOVA was employed for each infection assay screening on *Paramecium* and *Tetrahymena* using *E. albertii* and *E. coli* DH5α with various MOIs (0, 10, 100, 1000); relative gene expression of *astA* and *cdtB* with various growth temperatures (25 °C and 30 °C); inhibition test of CTxB; anti-GM3 antibody with various concentrations (0–5 µg/mL) and different MOIs (0, 10, 1000) to assess the survival of *T. thermophila;* and viable cells of *T. thermophila* on the LIVE/DEAD assay with various bacterial strains (*E. albertii*, *E. coli* pCold, and *E. coli* pCold-*cdtB*) at different time points (0.5 and 24 hpi). In addition, Dunnett’s multiple comparisons test was conducted following each one-way ANOVA or two-way ANOVA. Specifically, Tukey’s multiple comparisons test was applied for relative gene expression. A significant threshold of *p* < 0.05 was applied to the whole statistical set.

## 3. Results

### 3.1. Infection Assays for Paramecium and Tetrahymena with E. albertii

**Figure 1:**
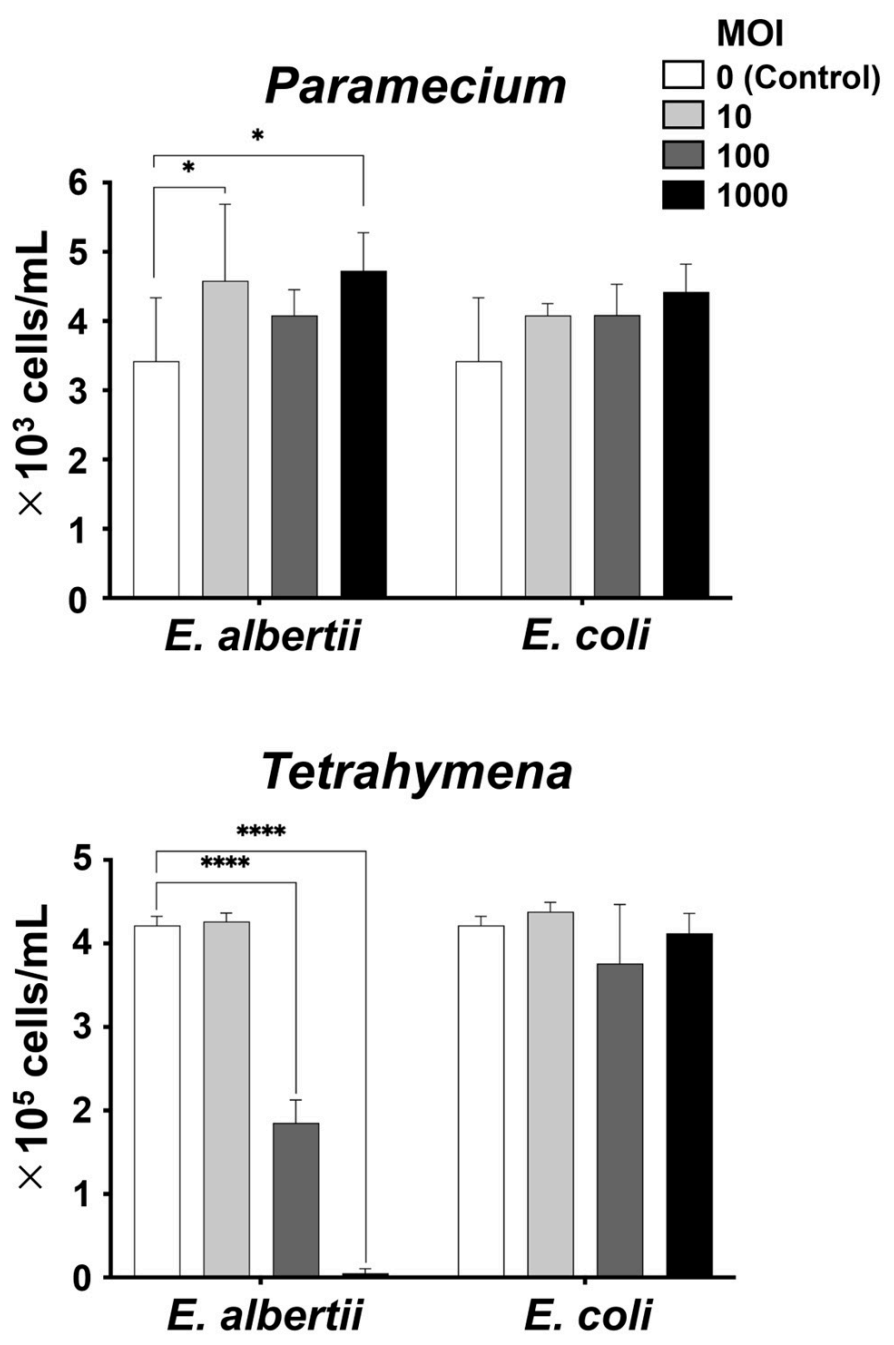
**Infection assays of *Paramecium* and *Tetrahymena* with *E. albertii* and *E. coli*.** Protist host cells were infected with *E. albertii* and *E. coli* DH5α at MOIs 10, 100, and 1000. The initial cell counts were 1 × 10^3^ cells/mL for *Paramecium* and 1 × 10^5^ cells/mL for *Tetrahymena*; viable cell counts are shown at 48 hpi. Data were obtained from duplicates of identical experiments, and statistically significant differences compared to the control are indicated by asterisks (* *p* < 0.05, **** *p* < 0.0001).

Figure 1 shows the initial screening of *E. albertii* cytotoxicity against different protist species. The figure presents *Paramecium* cell survival at MOIs 10, 100, and 1000 and in control at 48 hpi after *E. albertii* infection, with *E. coli* DH5α-infected *Paramecium* used for comparison. No cytotoxicity was observed to *Paramecium*, and the cell number tended to increase depending on the amount of bacteria administered because *E. albertii* and *E. coli* functioned as a food source. In contrast, *E. albertii* reduced *Tetrahymena* viable cell numbers at an MOI of 100 and induced complete cell death at an MOI of 1000 (**** *p*< 0.0001).

### 3.2. Analysis of Toxin-Associated Genes in E. albertii

**Figure 2:**
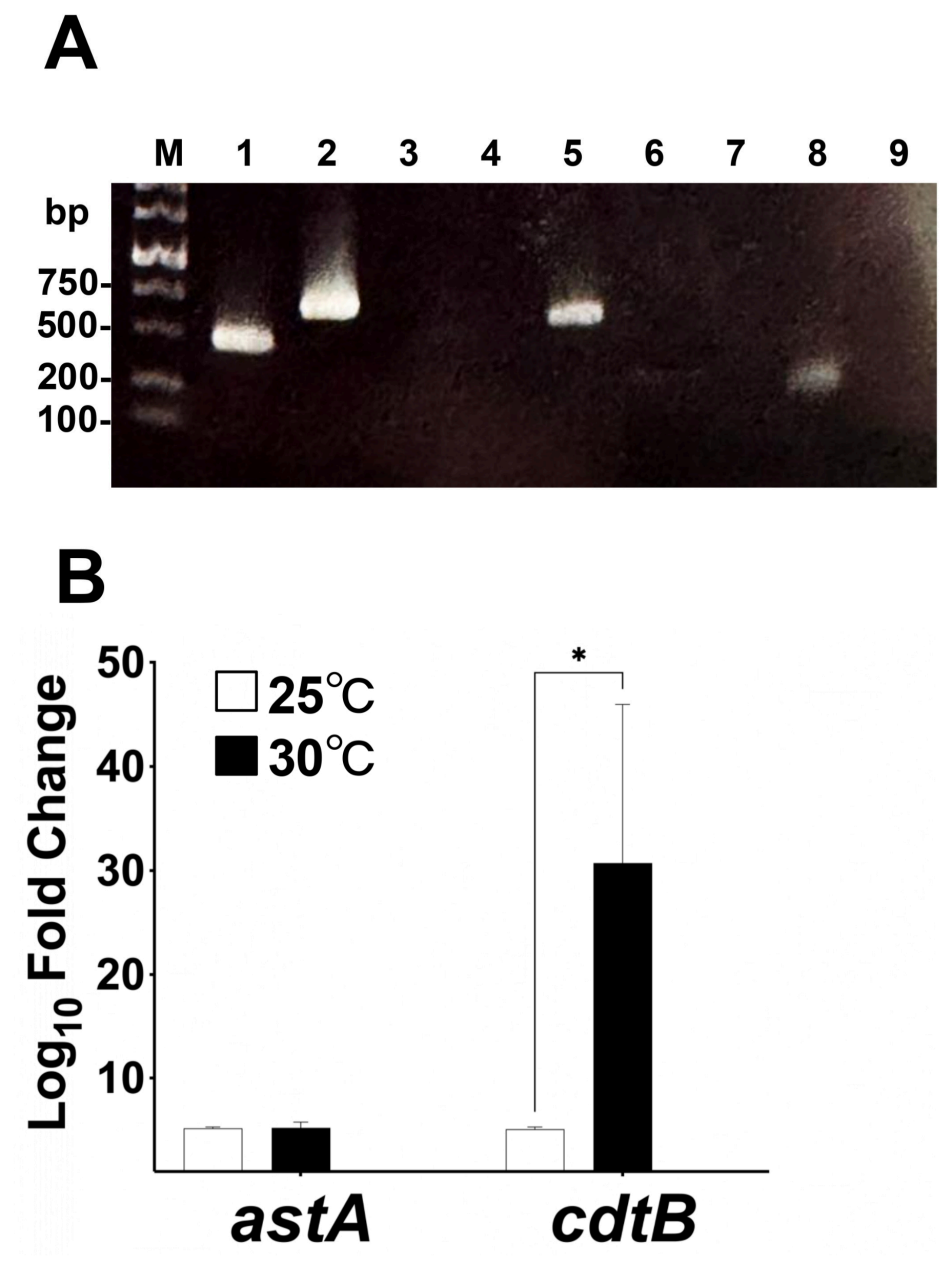
**PCR results for cytotoxicity-related genes in *E. albertii*.** (**A**) Lane M: molecular marker (bp), 1: *cdt*, 2: *cdt-IB*, 3: *cdt-IVB*, 4: *stx*, 5: *stx2f*, 6: *elt*, 7: *est*, 8: *astA*, and 9: negative control. (**B**) Relative expression level of *astA* and *cdtB* at each culture temperature compared to the level when *E. albertii* was cultured at 37 °C (defined as 1). The value was calculated by normalizing the target gene to the 16s rRNA gene as an endogenous control. Data were obtained from two identical experiments, and statistically significant differences among temperature differences are indicated by asterisks (* *p* < 0.05).

Toxin-associated genes in *E. albertii*, including *cdt*, *stx*, *elt*, *est*, and *ast* were screened by PCR (Figure 2A). It was found that this *E. albertii* strain carries *cdtB*, *stx2f*, and *astA*. Moreover, the relative expression of *astA* and *cdtB* genes for *E. albertii* tended to be higher in the optimum growth temperature of *Paramecium* (25 °C) and *Tetrahymena* (30 °C) in comparison with 37 °C (Figure 2B). In particular, *cdtB* showed the highest expression levels under 30 °C: *Tetrahymena* culture condition.

### 3.3. Confirmation of Ganglioside GM1 and GM3 Expression on Paramecium and Tetrahymena

**Figure 3:**
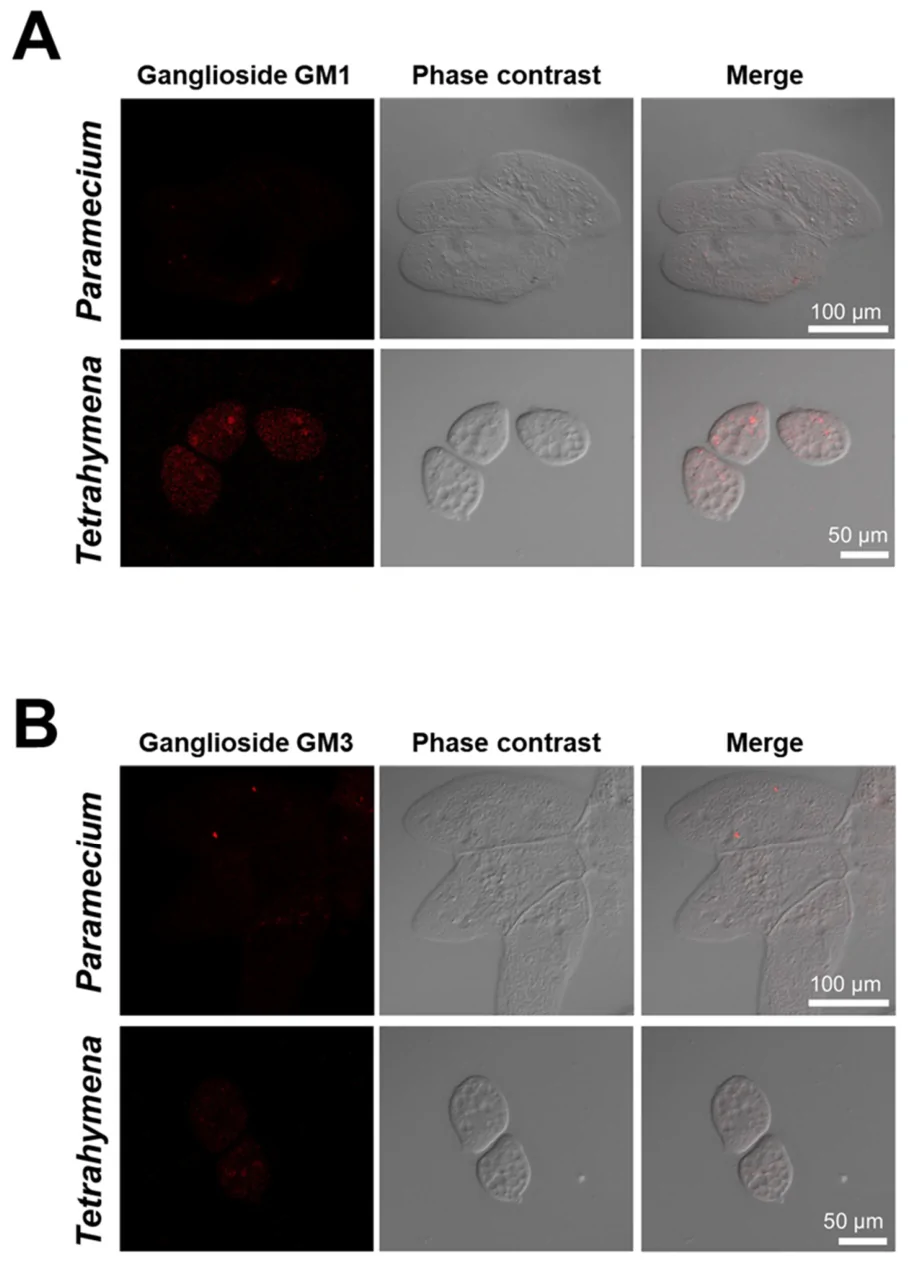
**Distribution of ganglioside GM1 and GM3.** The cellular distribution of ganglioside GM1 and GM3 in *Paramecium* (scale bar, 100 µm) and *Tetrahymena* (scale bar, 50 µm) was confirmed by fluorescence staining using CTxB (**A**) and anti-GM3 antibodies (**B**).

Next, we confirmed the distribution of ganglioside GM1 and GM3 in *Paramecium* and *Tetrahymena*, which are potential receptors for CDT. The fluorescently labeled CTxB probe did not react with *Paramecium* cells but showed a specific reaction with *Tetrahymena* cells, suggesting that the CDT receptor may be present in *Tetrahymena* (Figure 3A). Meanwhile, similar investigations using anti-GM3 antibodies revealed no specific or strong signal on the cell surfaces of either *Paramecium* or *Tetrahymena* (Figure 3B).

**Figure 4:**
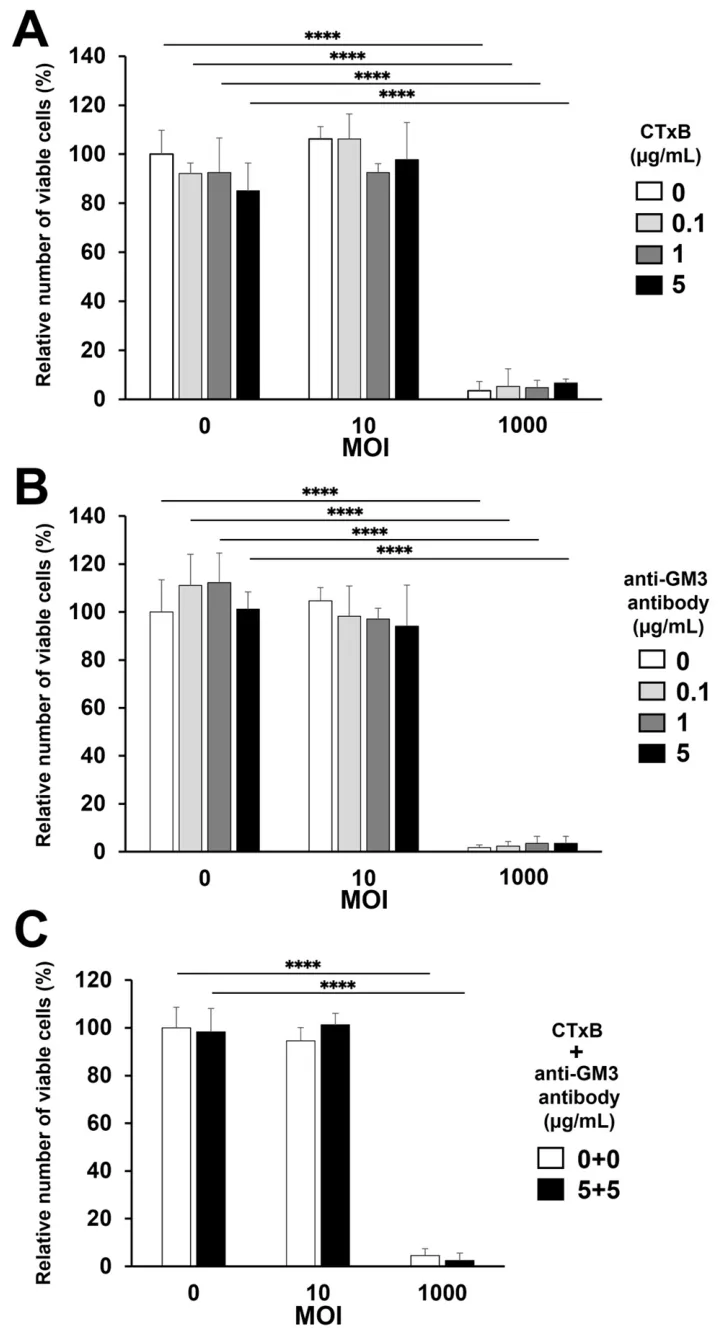
**An inhibition test using CTxB**. *T. thermophila* CU427 was infected with *E. albertii* at MOIs 0, 10, and 1000 with either CTxB (**A**) or anti-GM3 antibody (**B**) at concentrations of 0, 0.1, 1, and 5 µg/mL, or with both CTxB and anti-GM3 antibody at a concentration of 5 µg/mL (**C**). The viable cells were counted at 24 hpi. The relative number of viable cells is represented with the value for the uninfected (MOI of 0), untreated group set at 100%. Data were obtained from two identical experiments with duplicate samples, and statistical differences in MOI are presented by asterisks (**** *p* < 0.0001).

We therefore investigated whether CDT-dependent cytotoxicity of *E. albertii* against *T. thermophila* CU427 is reduced by antagonistic effects in the presence of CTxB. As a result, expected inhibitory effects were not observed, and no increase in CU427 survival rate was observed at any of the concentrations used (0–5 μg/mL; Figure 4A). Experiments using an anti-GM3 antibody yielded similar results as expected (Figure 4B), and no changes were observed even when CTxB and anti-GM3 antibody were administered simultaneously (Figure 4C).

### 3.4. Cytotoxicity Assay of T. thermophila CU427 Using Recombinant CdtB

**Figure 5:**
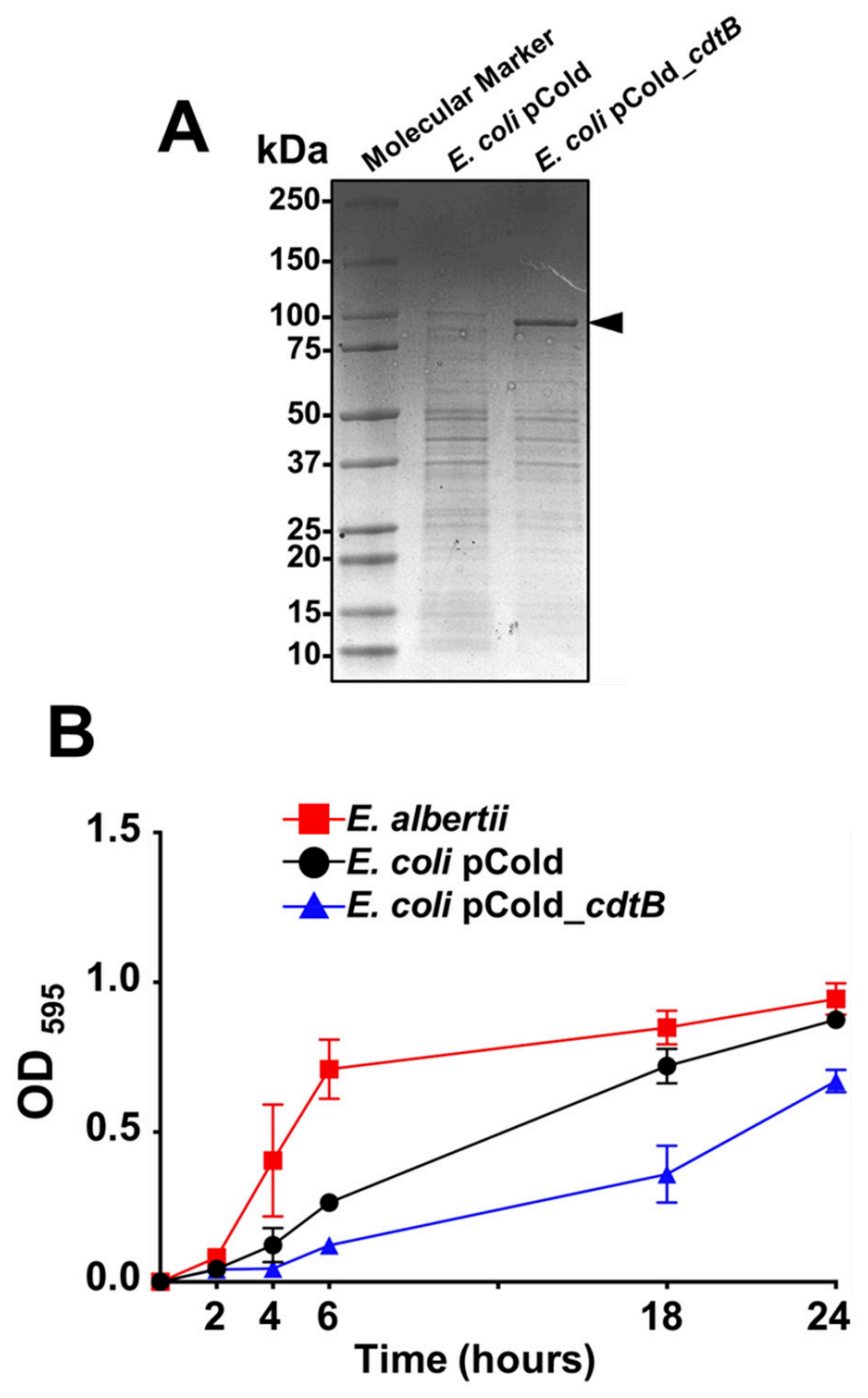
**Expression of CdtB in *E. coli*.** (**A**) Cell lysates were prepared from *E. coli* pCold and *E. coli* pCold_*cdt*B and subjected to SDS-PAGE. The arrowhead indicates the expressed recombinant CdtB protein. Since the recombinant CdtB is a fusion protein with a tag protein (52 kDa), its molecular weight is approximately 85 kDa. (**B**) Determination of bacterial growth rate. The data were presented as optical density numbers for *E. albertii*, *E. coli* pCold, and *E. coli* pCold_*cdt*B within 2, 4, 6, 18, and 24 h of time growth. Data were obtained from three identical experiments with triplicate samples.

To clarify CdtB involvement in *E. albertii* toxicity toward *Tetrahymena*, we focused on an approach using *E. coli* expressing recombinant CdtB. The *cdtB* gene was cloned from *E. albertii* into a pCold vector. Its expression in *E. coli* was confirmed by SDS-PAGE (Figure 5A). The growth rate of successful recombinant bacteria is presented in Figure 5B, where the growth of *E. coli* pCold_*cdtB* was slightly suppressed compared with *E. coli* pCold (empty-vector control), and the culture of *E. coli* pCold_*cdtB* reached an *OD*_595_ of 0.5 after 24 h (Figure 5B).

**Figure 6:**
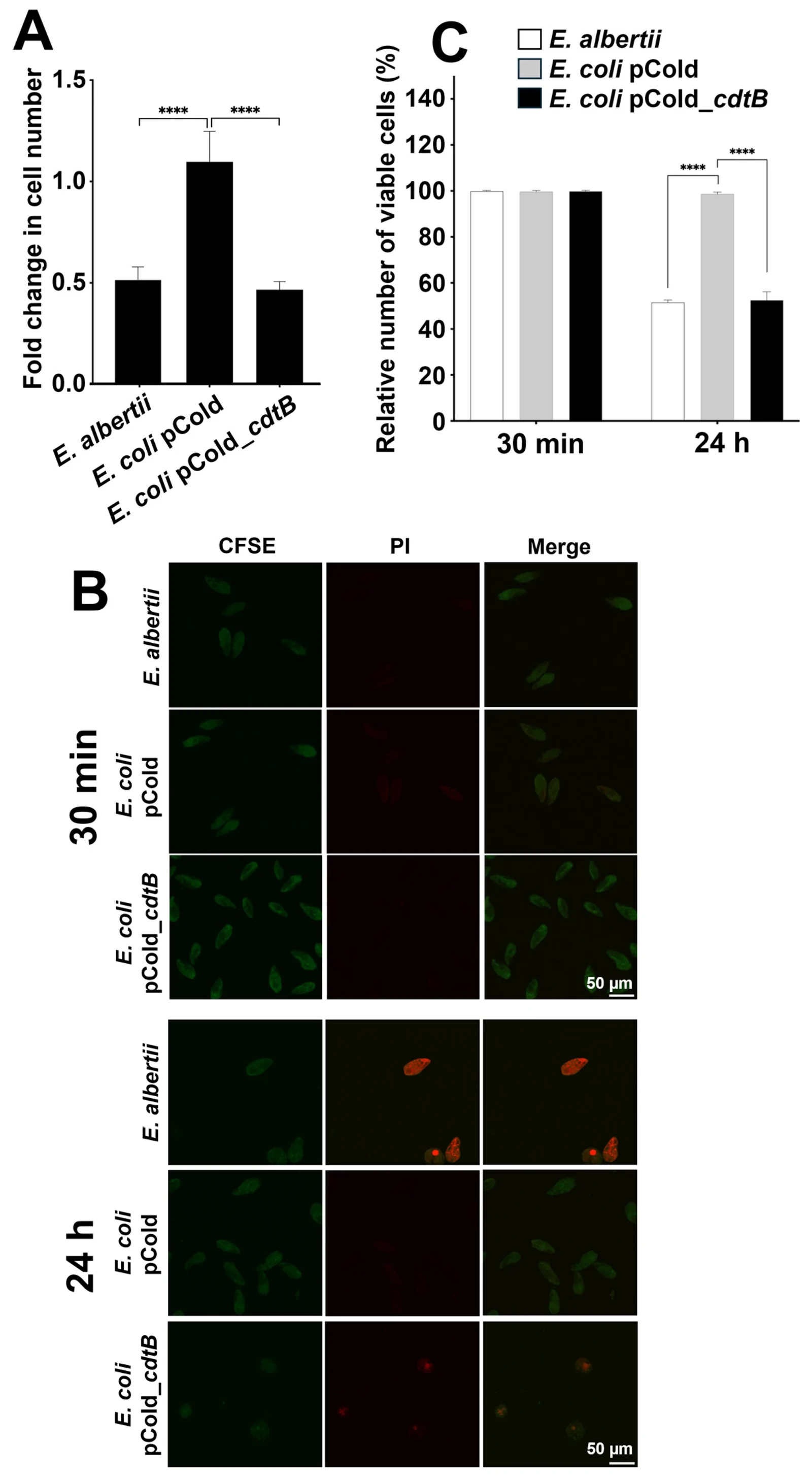
**Cytotoxicity assay in *T. thermophila* CU427 using recombinant CdtB.** (**A**) *T. thermophila* CU427 was infected with *E. albertii*, *E. coli* pCold, and *E. coli* pCold_*cdt*B at an MOI of 100. The viable cells were counted at 24 hpi and represented as the fold change in cell number with the value for the initial cell counts (2 × 10^5^ cells/mL) set at 1. Data were obtained from three identical experiments with triplicate samples, and statistically significant differences are indicated by asterisks (**** *p* < 0.0001). (**B**) *T. thermophila* CU427 morphology after infection with *E. albertii*, *E. coli* pCold, and *E. coli* pCold_*cdtB* (at an MOI of 100) at 30 °C. Cells were observed at 30 min (0.5 hpi) and 24 h (24 hpi) with CFSE/PI double staining. Scale bar, 50 µm. (**C**) Quantification of viable cell numbers based on CFSE/PI staining. The relative number of viable cells is calculated by randomly counting 100 cells. Data were obtained from three identical experiments with duplicate samples, and statistically significant differences are indicated by asterisks (**** *p* < 0.0001).

These strains were administered to CU427, and the viable cell numbers were evaluated at 24 hpi. Compared with the initial cell counts (2 × 10^5^ cells/mL), cells infected with *E. coli* pCold showed a slight increase. In contrast, cell numbers decreased by approximately half after infection with either *E. albertii* or CdtB-expressing *E. coli* DH5α (Figure 6A). Next, CFSE/PI double staining was used to verify the relationship between infection-induced cell number changes and cytotoxicity. During early infection (0.5 hpi), CU427 infected with all bacterial strains maintained normal size and motility and showed strong CFSE signals (Figure 6B, left). However, at 24 hpi, CU427 infected with *E. albertii* and *E. coli* pCold_*cdtB* eventually became a large number of nonviable cells with strong PI signals or abnormally circular shapes (Figure 6B, right). Quantification of viable cell numbers based on CFSE/PI staining revealed that viable CU427 infected with *E. albertii* and *E. coli* pCold_*cdtB* strain had decreased to approximately 50% at 24 hpi (Figure 6C). These results were very similar to those shown in Figure 1 and Figure 6A, where viable cell numbers were quantified based on morphology observed under a microscope.

## 4. Discussion

*Escherichia albertii*, a foodborne pathogen, causes gastroenteritis in humans, especially in infants and children. Abdominal distension, watery diarrhea, nausea, and fever are commonly reported clinical signs, with some bacteremia cases occurring in elderly patients [17,18]. Human *E. albertii* transmission may occur through contaminated water consumption. Therefore, water environment conditions markedly contribute to *E. albertii* survival [19,20]. Many aquatic protist predators may contribute to *E. albertii* dynamics, but the role of *T. thermophila* remains unclear. To begin testing this idea, we performed *E. albertii* infection assays in *Paramecium* and *Tetrahymena*, two close relatives within the Oligohymenophorea class. *Paramecium* ciliates are highly phagocytic protist predators and serve as symbiotic hosts for several Gram-negative bacteria [10,21,22]. *E. albertii* showed no specific relationship with *Paramecium* and was digested as prey, similar to many other bacteria, including *E. coli* [23]. In contrast, *E. albertii* showed strong cytotoxicity against *Tetrahymena* (Figure 1). Additionally, the observed differential susceptibility was not fully separated from differences in phagocytic uptake, phagosome processing, or intracellular bacterial persistence, which could therefore generate substantially different toxin exposures where it was part of a limitation of this study among species comparisons. As shown in Figure 2, our laboratory strain was positive for the *cdtB*, *stx2f* and *astA* genes embedded in *E. albertii*. Isolate screening from gastroenteritis outbreaks in Japan revealed that several *E. albertii* strains carried *cdt*, *stx* and *astA* toxin genes [24]. Although cytotoxicity studies of *E. albertii* with protist hosts remain unknown, cytotoxicity studies of toxins produced by *E. albertii* on cell lines have been reported several times. *E. albertii* harbors CDT-I, CDT-II, and Stx2f-induced Vero cell death [25], with a similar result of *E. albertii* harboring CDTs on different cell lines [26]. The present study showed that *astA* and *cdtB* gene expression increased under both 25 °C and 30 °C culture conditions compared with 37 °C (Figure 2B). In particular, *cdtB* expression increased approximately 40-fold when cultured at 30 °C, reflecting the temperature- or growth-phase-dependent regulation of *cdtB*, and this may be a factor contributing to cytotoxicity against *Tetrahymena*. Based on the differences in expression levels, our analysis shifted to focus specifically on *cdtB* rather than *astA.* Temperature shifts during *E. coli* growth (28–33 °C) can increase or decrease gene expression and affect transcriptional changes across functional classes, including cellular processes, DNA synthesis, and protein synthesis [27]. Stx is also an important toxin causing food poisoning in humans [28], but in our preliminary experiments conducted previously, EHEC carrying Stx showed no cytotoxicity against our *Paramecium* or *Tetrahymena* strain cells; thus, it was excluded from the analysis in this study.

CTxB intrinsically manipulates the membrane shape and binds ganglioside GM1 with high affinity in living-cell plasma membranes. After binding, CTxB diffuses extremely slowly and mediates endocytic uptake and retrograde secretory-pathway transport to the endoplasmic reticulum [29,30]. Beyond ganglioside GM1, ganglioside GM3 is the simplest membrane-bound glycosphingolipid in mammalian tissues; its abundance in nonneural organs supports bacterial toxin binding and may promote disease development. Collectively, gangliosides GM1 and GM3 may serve as primary routes for some bacterial enterotoxins, including CdtB, to reach their final intracellular pathways [8,9]. However, there are few reports on the membrane properties of these gangliosides in protists. *Trichomonas foetus* displays classical detergent-resistant membrane microdomains on its cell surface [31], and their localization in *Tetrahymena* and *Paramecium* is unknown. To investigate the possibility that ganglioside GM1 and GM3 work as important receptors for CdtB in *Tetrahymena*, we evaluated ganglioside GM1 and GM3 distribution in *T. thermophila* and, for comparison, in *P. caudatum.* As a result, a strong ganglioside GM1 distribution in *T. thermophila* was demonstrated. Thus, we hypothesized that CdtB of *E. albertii* may use ganglioside GM1 as a receptor and promote cell death. However, Figure 4A confirms that CTxB administration did not affect the 90% decrease in viable *T. thermophila* cell numbers at an MOI of 1000. Low ganglioside GM3 distribution in *T. thermophila* and *Paramecium* (Figure 3B) and the result that there are no changes in cytotoxicity after anti-GM3 antibody administration in *T. thermophila* suggest that ganglioside GM3 is also not involved in this cytotoxicity. However, the CTxB and particularly anti-GM3 antibodies used in this study may have had insufficient affinity for protist-cell gangliosides GM1 and GM3, resulting in no detectable cytotoxicity reduction. Further experiments using protist cells lacking these receptors would likely strengthen our results.

To clarify the role of CdtB in *E. albertii* cytotoxicity against *Tetrahymena*, we attempted to construct a *cdtB*-deletion strain; however, the mutant strain establishment was unsuccessful. Therefore, as an alternative analytical approach, we sought to construct a recombination protein of *cdtB* of *E. albertii* using a pCold vector. Although the recombinant strain showed delayed entry into the exponential growth phase, Figure 5A confirms the target CdtB protein expression. It is thus possible that the effects of CdtB overexpression on the bacteria are related to changes in bacterial physiology and growth. *E. albertii cdt* operons, which encode the three CDT subunits CdtA, CdtB, and CdtC, are frequently detected among *E. albertii* strains. CdtB works through DNase I activity and promotes DNA damage, resulting in cell death [18,32]. The heterotoxin CdtB requires CdtA and CdtC for host cell binding and subsequent CDT intoxication [8,33]. In human monocytic cells, CdtA and CdtC subunits are required for strong binding to gangliosides GM1 and GM3 and the initiation of full CdtB-mediated cytotoxicity [8]. Interestingly, this study showed that *E. coli* DH5α expressing *E. albertii* CdtB alone also exhibited some cytotoxicity against *T. thermophila* (Figure 6). Because most *E. albertii* cells are ingested by *Tetrahymena* during early infection and persist within phagosomes, we hypothesized phagosome-secreted CdtB may act on host cells. A *Tetrahymena*-specific CdtB receptor, absent in *Paramecium*, may exist on the phagosome membrane. The corresponding activity mechanism may be unique to *Tetrahymena* and independent of CdtA and CdtC. To fully clarify these findings, subsequent trials with modified parameters such as measuring DNA damage [34] or related cellular responses in *Tetrahymena* [35] will be essential to exclude non-specific effects associated with recombinant protein expression, altered bacterial physiology, or other stress-related mechanisms.

This study has several limitations: First, only a single strain of *E. albertii*, *T. thermophila* and *Paramecium* were used in this study, which may limit the broad generalization of protist–host interaction. Second, fixed-cell staining only confirms the presence of GM1 and does not validate inhibitor accessibility or affinity during intoxication. Third, the LIVE/DEAD assays were only carried out without any following DNA damage or nuclease activity in the *Tetrahymena*. Therefore, all these limitations may affect the interpretation and generalizability of these findings.

Although *E. albertii* shares almost similar properties with enteropathogenic *E. coli*, its survival within *T. thermophila* remains a distinctive fate [14]. Our findings indicate that *E. albertii*-encoded CdtB is involved in the cytotoxicity in *T. thermophila* cells and does not require GM1 or GM3 binding. The observed *E. albertii* cytotoxicity indicates that *E. albertii* may have a limited capacity to use *Tetrahymena* as a natural host or vector. Although bacterial or host strains and interaction conditions may influence this phenomenon, limiting broad generalization, the findings imply unique and diverse protist–bacteria relationships in aquatic environments. We provide the first deeper insights into CdtB toxin properties in *E. albertii* toward the viability of *T. thermophila*. CdtB may trigger cytotoxicity in *T. thermophila* by binding to receptors other than gangliosides GM1 and GM3, inducing cell damage without CdtA- and CdtC-mediated early attachment.

## Figures and Tables

**Table 1:** Primer list.

Gene		Sequence 5′-3′	Product Size (bp)	References
*cdt*	F	GCTTAACTGGATGATTCTTG	449	[16]
	R	CTATTTCCCATCCAATAGTCT		
*cdt-IB*	F	GATTTTGCCGGGTATTTCT	640	[16]
	R	TCAAGAACACCACCACTG		
*cdt-IVB*	F	ACCATCTTCAGCTACACTA	286	[16]
	R	GCTCCAGAATCTATACCT		
*stx* (excluding *stx2f*)	F	GAGCGAAATAATTTATATGTG	518	[16]
	R	TGATGATGGCAATTYARTAT		
*stx2f*	F	GAACGGAATAACTTATATGTT	518	[16]
	R	GTTGAGTGGCAATTAAGGAT		
*elt*	F	AGCAGGTTTCCCACCGGATCACCA	130	[16]
	R	GTGCTCAGATTCTGGGTCTC		
*est*	F	ATTGTCTATTTTTMTTTCTGTATTRTCTTT	183	[16]
	R	GGATTACAACAAAATTCACAGCAG		
*astA*	F	GCCATCAACACAGTATATCC	106	[16]
	R	GAGTGACGGCTTTGTAGTCC		
CdtB for pCold	F	ATTCAAGCTTGTCGACATGAAAAAATATATTATATCTCT	-	This study
	R	TAGACTGCAGGTCGATTACCGTCTGGATACGCC		
16s rRNA (for qPCR)	F	TAAGGAGACTGCCGGTGACA	-	This study
	R	GGCCATTGTAGCACGTGTGT		
*cdtB* (for qPCR)	F	AGGAGGACGACCATTGCTTG	-	This study
	R	TTAAGCGCCTGGTGTACTGG		
*astA* (for qPCR)	F	GATTTTAGCCGCGCCGATTT	-	This study
	R	ACCTTCTTTCTCCAGCACCG		

## Data Availability

Data are available on request to the corresponding author.
